# Comparative Inference of Duplicated Genes Produced by Polyploidization in Soybean Genome

**DOI:** 10.1155/2013/275616

**Published:** 2013-10-09

**Authors:** Yanmei Yang, Jinpeng Wang, Jianyong Di

**Affiliations:** ^1^College of Science, Hebei United University, Tangshan, Hebei 063009, China; ^2^Center for Genomics and Computational Biology, School of Life Sciences, Hebei United University, Tangshan, Hebei 063009, China

## Abstract

Soybean (*Glycine max*) is one of the most important crop plants for providing protein and oil. It is important to investigate soybean genome for its economic and scientific value. Polyploidy is a widespread and recursive phenomenon during plant evolution, and it could generate massive duplicated genes which is an important resource for genetic innovation. Improved sequence alignment criteria and statistical analysis are used to identify and characterize duplicated genes produced by polyploidization in soybean. Based on the collinearity method, duplicated genes by whole genome duplication account for 70.3% in soybean. From the statistical analysis of the molecular distances between duplicated genes, our study indicates that the whole genome duplication event occurred more than once in the genome evolution of soybean, which is often distributed near the ends of chromosomes.

## 1. Introduction

There is an important economic and scientific value of study on soybean genome. First, soybean (*Glycine max*) is one of the most important crops for producing protein and oil. Second, it has the capacity to fix nitrogen which is one of the major problems of life science. Biological nitrogen fixation provides all the plants with 75% of nitrogen, which plays an important work in the practical production. The completions of genome sequencing in legumes provide new ideas for the studying of symbiotic nitrogen fixation on the genome level [1].

Large-scale duplication events have been considered important for the evolution of many organisms. About 70% of angiosperm evolution has experienced one or more polyploidization events. Polyploidy is a widespread and recursive phenomenon during plant evolution, and it could generate massive duplicated genes which is an important resource of genetic innovation [2–6]. This process greatly increases the complexity of the plant and improves the adaptive capacity to new environments.

Soybean is a well documented paleopolyploid [7]. Despite some analysis of soybean [8], the in-depth study of evolution after polyploidization is also needed. For comparative genomics analysis, this paper aims to study the evolution laws of duplicated genes in soybean after polyploidization. Our study identifies significant duplicated genes on the evolutionary of the two species, *soybean *and *Arabidopsis thaliana*, and indicates that the whole genome duplication (WGD) event occurred more than once in the genome evolution of soybean.

## 2. Materials and Methods

### 2.1. Sequence Data

Soybean (*Glycine max*) [1] and *Arabidopsis thaliana *[9] genome sequences were downloaded from Plant Genome Duplication Database (http://chibba.agtec.uga.edu/duplication/). 66210 and 27379 DNA and the corresponding protein sequences of *Arabidopsis* and soybean are obtained, respectively. And the locations of each gene on the chromosome are also identified.

### 2.2. Gene Collinearity Inference

**Figure 1:**
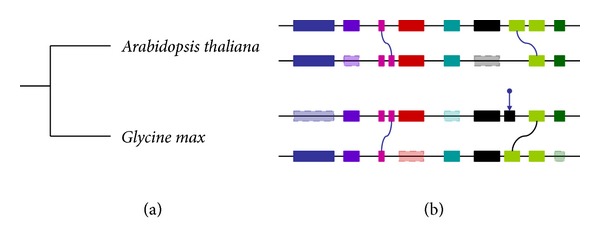
(a) Phylogenetic trees for *Arabidopsis thaliana* and *Glycine max*. (b) Sketch map of collinear genes in blocks. A pair of horizontal lines which have the same color represent one collinear DNA fragment in one species. Horizontal lines with different colors represent collinear DNA fragment from different species in phylogenetics tree. Different genes denoted by rectangles have different colors, and the collinear homologous genes denoted by rectangles have the same colors. Rectangles surrounded by imaginary line represent the gene loss events. The blue curve shows the gene duplication, and the blue arrows indicate the gene insertion.

In this paper, *Arabidopsis* and soybean are selected as our research objects, which have common polyploid ancestors. Using model organisms *Arabidopsis* genome as a reference, the collinearity method is applied to infer homologous genes in the soybean genome (Figure 1).

First, the similarity between soybean and *Arabidopsis thaliana *is predicted by running BLASTP (*E*_−_value < 1 × 10^−5^). And then the syntenic regions are visualized by MCscan. The built-in scoring scheme for MCscan [10, 11] is min(−log⁡10 *E*_−_value, 50) for every matching gene pair and −1 for each 10 kb distance between anchors, and blocks that had scores >300 were kept. The homology gene blocks are determined by searching the maximal match of the result by MCscan; then, the algorithm of searching the really paralogs is listed as follows:

1. process the aligns in output data of the MCscan to obtain the size of collinear genes blocks, such as the size of blocks of collinear genes pairs. A-B is *N*_1_, and A-C is *N*_2_;
2. compare the size of the collinear genes blocks with other different genes;
3. identify the true paralogs produced by WGD. If *N*_1_ > *N*_2_, then, the A-B is a pair of paralogs gene.

The above process is implemented on Linux by the perl programming language.

## 3. Results

### 3.1. Identify Similarity between Genes

**Figure 2:**
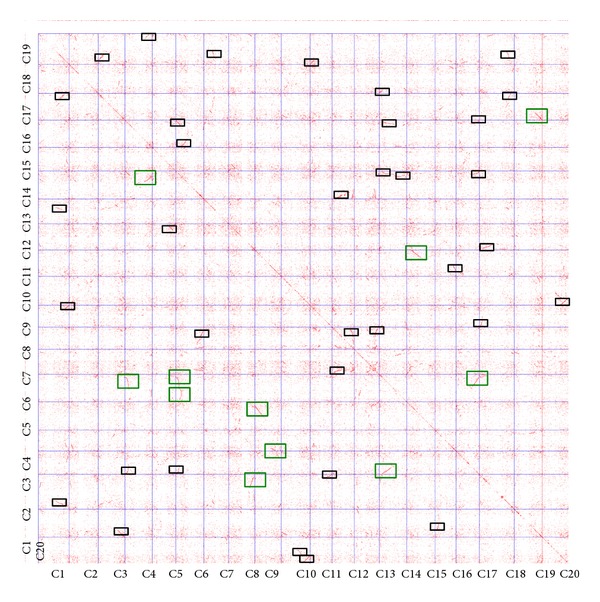
The dot plot of soybean genome.

Dot plots can visualize the similarity between two proteins or nucleic acid sequences; when the sequences match at the same location on the plot, a dot is drawn at the corresponding position [12]. The DNA dot plot of soybean genome in Figure 2 shows regional self-similarity: (1) *X*-axis aligned the 20 soybean chromosomes from left to right and *Y*-axis aligned the sequence from top to bottom; (2) based on homologous information by Blast and the location of genes on chromosomes, each pair of homologous genes in the two-dimensional plan can be formed on a point; (3) a series of consecutive points in the plane would constitute a different line. From the figure, we can find that the soybean genome contains extensive duplicated genome.

### 3.2. The Scale of Genome Duplication in Soybean

The level of gene duplication in soybean genome is significant. We find that 23,269 pairs of paralogs, including about 46,538 genes, could account for 70.3% of total genes in soybean genome. All these genes are duplicated genes produced by the whole genome duplication. In total, 47 duplicated blocks located on all 20 chromosomes are identified, in which the largest block contains 1070 pairs of paralogs, 2140 genes, which are mainly distributed in the chromosomes 3 and 19 of soybean; the smallest block contains 5 pairs of paralogs, 10 genes, which are mainly located on chromosome 5 and 6.

### 3.3. Multiple Gene Duplication Events

**Figure 3:**
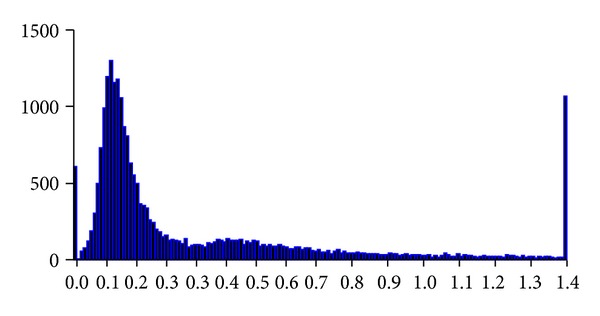
Synonymous distance between paralogs in soybean.

A synonymous substitution is the evolutionary substitution of one based for another in an exon of a gene coding for a protein, such that the produced amino acid sequence is not modified. A nonsynonymous substitution results in a change in amino acid [13]. Applying NG-86 model, the values of synonymous substitution Rate (*K*_*s*_) and nonsynonymous substitution rate (*K*_*a*_) are calculated between homologous genes in soybean. Synonymous distances between paralogs in soybean are shown in Figure 3. Form the figure, we can see two obvious peaks; the *K*_*s*_ is about 0.15 and 0.42, respectively. In addition, the *K*_*s*_ which is about 520 pairs of paralogs is almost 0, and about more than 1200 pairs of paralogs are greater than 1.5. The data showed that the multiple of massive gene duplication event may occur in the genome of soybean. The values of synonymous substitution rate (*K*_*s*_) between duplicated genes are very small. The results showed that the duplicated genes were produced by recent duplication event, which also showed the existence of the ongoing duplication event in the soybean genome.

### 3.4. Whole Genome Duplication and Gene Physical Location

The distribution of duplicated genes on the chromosome produced by the whole genome duplication may be related to its physical location. To infer the potential rule here, we divide all the soybean genes into two categories: (1) duplicated genes produced by WGD and (2) nonduplicated genes. Firstly, computing the distance of each duplicated genes and nonduplicated genes to chromosome ends and, secondly, comparing the average distance of duplicated genes and nonduplication genes to the chromosome ends, we applied the paired two-sample analysis. For these two sets of distance *D*_*n*_ (to the ends of chromosomes), we can state that the null hypothesis is that the means between the two distance sets are equal, respectively. The two-sample test is a hypothesis test for answering questions about the mean where the data are collected from two random samples of independent observations [14]. For a duplicated gene in soybean, if the existence of distance *D*_*n*1_ of *N*_1_ paired duplicated genes and distance *D*_*n*2_ of *N*_2_ nonrepetitive genes which calculates the mean of *N*_1_ and *N*_2_ is *x*_1_ and *x*_2_, test statistic of *Z*-test is analyzed to test whether the difference between two means is equal to a number; statistics are

(1) $$\begin{array}{c} Z=\frac{\left(x_{1}-x_{2}\right)-d_{0}}{\sqrt{\delta_{1}^{2}/n_{1}+\delta_{2}^{2}/n_{2}\;}}. \end{array}$$

Assume that *d*_0_ is the mean difference of *D*_*n*1_ (the mean of duplicated genes) and *D*_*n*2_ (the mean of nonduplicated genes), where the mean difference is assumed to be 0; *δ*_1_, *δ*_2_ are the variance of the two samples. Our analysis found that duplicated genes are often located near the chromosome ends, and whole genome duplication and gene physical location are shown in Table 1. The average distance of duplicated genes to chromosome ends in soybean is 6.6 Mbp (*P*_value = 0 ≫ 0.05), while the average distance of all genes is 11 Mbp (*P*_value = 0 ≪ 0.05). In soybean, about 40% of duplicated gene pairs are distributed close to the ends of chromosomes where the distance is 4 Mb, as shown in Table 1.

As shown above, the physical location of genes on the chromosome is closely related to whole gene duplication, while duplicated genes produced by WGD are distributed close to the ends of chromosomes. This finding is reasonable because of the threefold judgment. First, there is a large number of redundancies of more conservative sequence far from centromere; second, gene collinearity is often found in areas of high density; third, the original copies are often near the ends of chromosomes. DNA rearrangement and gene codon mutation reduced the similarity between sequences, resulting in the emergence of new gene sequences, which may have new functions to support the organism to adapt to the new environment.

## 4. Discussion

Our studies suggest that the whole genome duplication event occurs more than once in the genome evolution of soybean. It is proofed that soybean has undergone polyploidy events. The scale of genome duplication is obtained by our new method. Based on the collinearity method, the duplicated genes which cover 70.3% of the soybean genome identified are produced by the whole genome duplication. Furthermore, these duplicated genes are often distributed near telomeres. One of the reasons is that the original copies are often near the ends of chromosomes. Our findings also raise the possibility that the ongoing duplication event exists in the soybean genome.

Duplicated genes are important resources of genetic innovation. Identifying the scale of gene multiplication in genome can provide important information for the study of genetics. DNA rearrangement and gene codon mutation reduced the similarity between sequences, resulting in the emergence of new gene sequences. Although an increasing number of reports have revealed the phenomenon of gene duplication, the origins and mechanisms of duplicated gene and the evolutionary fate of duplicated gene still lack sufficient research. Revealing the contribution of gene and genome duplications to the evolution and domestication of these plants is a significant research; we hope that our study will promote the research in the field.

## Figures and Tables

**Table 1:** Whole gene duplication and gene physical location.

Distance to telomere	<4 Mbp	4–8 Mbp	8–12 Mbp	12–16 Mbp	16–20 Mbp	>20 Mbp
Genes	19660	15841	11937	8060	6112	4601
Duplicated genes	18311	13115	8027	3774	2075	1236
